# Comparison of Muscle Density in Middle-Aged and Older Chinese Adults Between a High-Altitude Area (Kunming) and a Low-Altitude Area (Beijing)

**DOI:** 10.3389/fendo.2021.811770

**Published:** 2021-12-24

**Authors:** Xingli Liu, Ling Wang, Meng Gao, Gang Wang, Kai Tang, Jin Yang, Wei Song, Jingsong Yang, Liang Lyu, Xiaoguang Cheng

**Affiliations:** ^1^ Faculty of Life Science and Technology, Kunming University of Science and Technology, Kunming, China; ^2^ Medical School, Kunming University of Science and Technology, Kunming, China; ^3^ Department of Radiology, The First People’s Hospital of Yunnan Province, Kunming, China; ^4^ Department of Radiology, The Affiliated Hospital of Kunming University of Science and Technology, Kunming, China; ^5^ Department of Radiology, Beijing Jishuitan Hospital, Beijing, China

**Keywords:** altitude, muscle density, muscle area, older adults, computed tomography

## Abstract

**Background and Purpose:**

A high-altitude environment was known to have a negative effect on bone and lead to a higher incidence of hip fracture. However, the dependence of muscle composition on altitude is unclear. Thus, we aimed to compare muscle density and area in plateau and low altitude area and to determine the effect of the altitude on these outcomes.

**Methods:**

Community dwelling adults over 60 years old living in Beijing (elevation 50 m; 300 subjects,107 men and 193 women) or Kunming (elevation 2000 m; 218 subjects,83 men and 135 women) for more than 10 years were enrolled. Quantitative CT was performed in all subjects and cross-sectional area and attenuation measured in Hounsfield units (HU) were determined for the trunk, gluteus, and mid-thigh muscles.

**Results:**

Compared to Beijing, Kunming adults were slimmer (Beijing men *vs* Kunming men: 25.08 ± 2.62 *vs* 23.94 ± 3.10kg/m^2^, *P*=0.013; Beijing women *vs* Kunming women: 25.31 ± 3.1 *vs* 23.98 ± 3.54 kg/m^2^, *P*= 0.001) and had higher muscle density in the L2-trunk and gluteus maximus muscles after adjustment for age and BMI (L2-trunk muscles: Beijing men 29.99 ± 4.17 HU *vs* Kunming men 37.35 ± 4.25 HU, *P*< 0.0001; Beijing women 27.37 ± 3.76 HU *vs* Kunming women 31.51 ± 5.12 HU, *P*< 0.0001; Gluteus maximus muscle: Beijing men 35.11 ± 6.54 HU *vs* Kunming men 39.36 ± 4.39 HU, *P*= 0.0009; Beijing women 31.47 ± 6.26 HU *vs* Kunming women 34.20 ± 5.87 HU *P*=0.0375). Age was similar in both cohorts and no differences were observed in the gluteus medius and minimus muscle or the mid-thigh muscle, either in the area or density.

**Conclusions:**

Compared with Beijing, the adults in Kunming had higher muscle density of the gluteus maximus and L2 trunk muscles, showing that living at a higher altitude might be beneficial to muscle quality.

## Introduction

Hip fractures are the most severe type of osteoporotic fracture and in an aging society have become a heavy public health burden. Recent studies based on large sample sizes have found that the incidence rate of hip fracture is associated with altitude, with higher altitude areas having higher hip fracture rates than lower altitude (1). The underlining mechanisms are still unknown, but hypoxia may play an important role. Hypoxic environments have been shown to influence body composition (eg. reductions in body weight, fat-free mass, fat mass, muscle mass, and/or body water) (2–4). Weight loss has been widely reported in hypoxic chamber experiments and after sojourns at high altitudes (5–7). Some studies have found that skeletal muscle mass decreased with increasing altitude as the hypoxic environment accelerated the decomposition of skeletal muscle and inhibited protein synthesis (8, 9). Muscle weakness is a key factor in the increased risk of falls and might also play a significant role in the increased risk of hip fracture (10, 11). Muscle density, which has been proved to be an important indicator for the evaluation of muscle function, correlates well with muscle strength and physical performance (12). However, few studies have explored the effects on skeletal muscle of hypoxic conditions at high altitude, and the existing findings are mostly based on bioimpedance analysis (BIA) or dual-energy X-ray absorptiometry (DXA) acquisition (8, 13). More precise techniques and data were needed for further validation.

Thus, this study aims to compare the muscle characteristics of people living in Beijing (elevation: 50 meters above sea level) and in Kunming (elevation: 2000 meters above sea level) by quantitative CT to explore the effect of altitude on muscle in middle-aged and older adults. We hypothesized that people in Kunming (high altitude) have poor muscle quality compared to the Beijing population (low altitude).

## Materials and Methods

### Study Participants

Independently living community-dwelling adults residing within the region of Beijing Jishuitan Hospital and the First People’s Hospital of Yunnan Province were recruited using convenience sampling, respectively. 300 subjects in Beijing (107 men and 193 women) were enrolled between March 2017 and July 2017, and 218 subjects in Kunming (83 men and 135 women) were enrolled between March 2021 and July 2021. All participants were aged 60 years or older and had been living in either Beijing or Kunming for at least 10 years. Exclusion criteria were as follows: 1. Inability to move independently; 2. Non-osteoporotic pathologic fractures; 3. Deformity of the lumbar spine and hip joint; 4. Tumors treated with radiotherapy or chemotherapy; 5. Patients with metallic implants *in vivo*; 6. Other serious or life-threatening diseases. The study was approved by the local ethics committees in Beijing and Kunming respectively [approval number: 201512-02 (Beijing) and KHLL2021-KY056 (Kunming)]. Informed consent was obtained from each participant.

### CT Acquisition

Lumber, hip, and midthigh CT imaging together with a Mindways calibrated CT/QCT acquisition phantom (Mindways Software Inc, Austin, TX, USA) was performed for all study participants. CT scanner information was as follows: Kunming cohort: a third generation dual-source CT scanner (Siemens Force CT, Siemens Healthcare, Germany); Beijing cohort: Toshiba Aquilion CT scanner (Toshiba Medical Systems Division, Tokyo, Japan). All scans were acquired in the supine position. Scan parameters for all CT scans were 120 kVp, 150 mAs, slice thickness: 1.5mm, Pitch 1.5mm, 512 x 512matrix.

### Muscle Assessments

**Figure 1:**
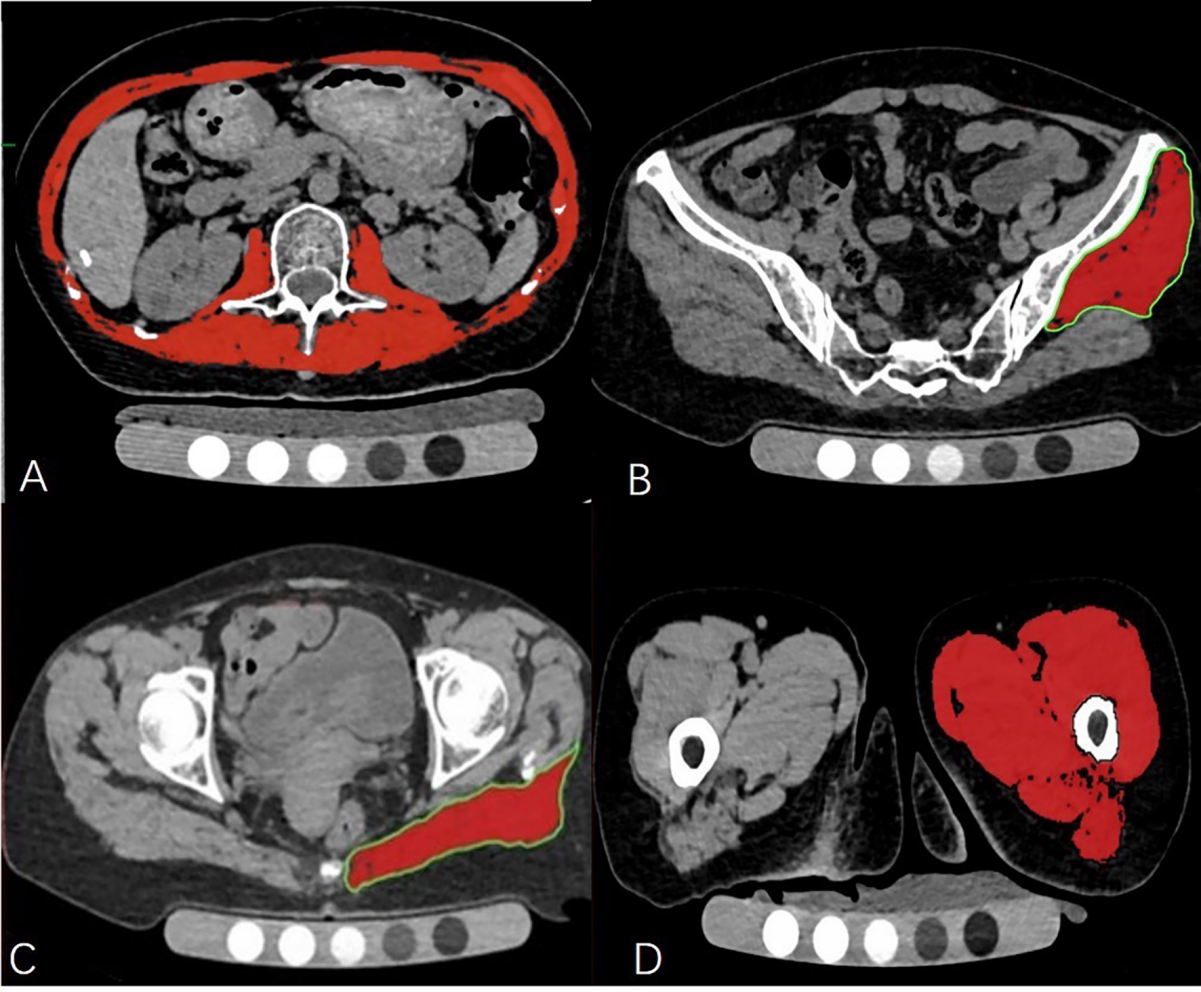
The cross-sectional level of muscle measurement **(A–D)**. Measurement of the trunk muscle at mid—L2 level **(A)**; Measurement of the left gluteus medius and minimus muscle at the 3rd sacral (S3) level **(B)**; Measurement of the left gluteus maximus at the level of the greater trochanter of the femur **(C)**; Measurement of the left mid-thigh muscle group **(D)**.

The density and axial area of trunk muscle at the L2 level, left side of gluteus maximus muscle, gluteus minimus & medius muscle and mid-thigh muscle were each measured on a single slice. The criteria of measurement section position were as follows: 1. trunk muscle: at the level of the second lumbar vertebra transverse process; 2. left gluteus maximus muscle: at the level of greater trochanter of the femur gluteus; 3. left gluteus minimus & medius muscles: at the 3rd sacral (S3) level; 4. left mid-thigh muscles: at the level of 3cm below the lesser trochanter (**Figure 1**).

OsiriX software (Lite version 10.0.2; Pixmeo, Geneva, Switzerland) was used for muscle analysis. Firstly, The Dicom images of the participant were imported into Orisix software. Secondly, muscle segmentation was performed manually using the ‘pencil’ tool to outline muscle contours. Thirdly the ‘GrowRegion (2D/3D Segmentation)’ tool was used to semiautomatically select skeletal muscle regions within our preset HU intensity thresholds (-30 to150HU) (14). Within the resulting muscle region of interest (ROIs), a threshold of -29 HU was applied to distinguish muscle tissue from fat (15). Then the muscle CSA and density of the selected ROI were displayed on the screen. To minimize the resulting error caused by layer selection, all the muscle measurements were performed by the same investigator who had received professional training in CT muscle imaging before the analysis.

The HU values of water equivalent materials of the European spine phantom (ESP-128) were measured and used for the cross-calibration of muscle attenuations of the two CT scanners.

### Statistical Analyses

All statistical analyses were conducted using SPSS (Version 22, IBM) and MedCalc (Version 18, MedCalc). Continuous variables were presented as mean ± standard deviation. Spearman correlation testing was used to analyze the correlation of muscle density with age and BMI. Independent t-test was selected for normal distribution data while the nonparametric test (Mann-Whitney U) was applicable to non-normal distribution data to compare muscle size and density between Beijing and Kunming participants. Comparisons among groups were performed using variance analysis and linear regression models. All models were adjusted for BMI and age. The area of the gluteus minimus & medius muscle was not included in the analysis, as the inconsistent measurement slices in the Beijing population introduced a 10% bias, which was described in a previous study (12). A *P* value of less than 0.05 indicated statistical significance.

## Results

### Basic Information and Muscle Difference Between Beijing and Kunming Group

Men and women in the Kunming and Beijing cohorts were well matched for age (**Table 1**). However, compared to the Kunming cohort, both men and women in the Beijing cohort had a statistically significantly higher BMI. Men in the Beijing cohort, but not the women, had a statistically larger waist circumference compared with the Kunming cohort. Unexpectedly, a higher muscle density in the gluteus maximus and L2-trunk muscles was observed in the Kunming group (gluteus maximus muscle: Beijing *vs* Kunming *P*_men_<0.0001, *P*_women_=0.0002; L2-trunk muscles: Beijing *vs* Kunming *P*_men_<0.0001, *P*_women_<0.0001) (**Table 1**).

**Table 1:** Difference of variables between Beijing and Kunming group.

	Men			Women		
	BeiJing (n = 107)	KunMing (n = 83)	*P* value	BeiJing (n = 193)	KunMing (n = 135)	*P* value
**Age (years)**	69.6 ± 6.63	67.9 ± 5.75	0.11	67.68 ± 5.77	66.9 ± 5.79	0.15
**BMI (kg/m^2^)**	25.08 ± 2.62	23.94 ± 3.10	**0.013**	25.31 ± 3.08	23.98 ± 3.54	**0.001**
**G.MaxM density (HU)**	35.11 ± 6.54	39.36 ± 4.39	**< 0.0001**	31.47 ± 6.26	34.20 ± 5.87	**0.0002**
**G.MaxM area (cm^2^)**	43.11 ± 7.9	44.67 ± 7.4	0.2802	37.27 ± 6.28	36.85 ± 6.15	0.5789
**G.Med/MinM density (HU)**	42.73 ± 4.0	43.48 ± 3.84	0.1355	41.11 ± 4.32	40.25 ± 4.48	0.2017
**Midthigh muscle density (HU)**	46.04 ± 3.64	46.57 ± 2.58	0.2419	43.49 ± 3.85	44.09 ± 3.17	0.2698
**Midthigh muscle area (cm^2^)**	123.55 ± 22.23	120.14 ± 18.85	0.1344	93.15 ± 14.51	93.13 ± 13.33	0.9769
**L2 trunk muscle density (HU)**	29.99 ± 4.17	37.35 ± 4.25	**< 0.0001**	27.37 ± 3.76	31.51 ± 5.12	**< 0.0001**
**L2 trunk muscle area (cm^2^)**	125.90 ± 18.01	125.60 ± 20.14	0.9327	90.35 ± 13.40	88.76 ± 11.96	0.5232
**Waistline (cm)**	89.93 ± 8.04	85.57 ± 8.40	**0.0005**	84.78 ± 8.44	85.84 ± 9.11	0.1885

Data presented as (mean ± SD). The values of P < 0.05 were marked in bold.

### Effects of Age and BMI on Muscles

Muscle density and area were associated with age or/and BMI (**Table 2**). Except for the gluteus maximus muscle of the male Beijing group, muscle density was correlated with age. However, the gluteus minimus and mid-thigh muscle densities were not significantly associated with BMI. The density differences of L2 trunk muscle and gluteus maximus muscle between subgroups were significant, no matter whether the data were adjusted for age or BMI. A density difference of gluteus medius and minimus muscles between women was found after adjusting for both age and BMI (*P*=0.0116). In addition, we noticed that only the area of the gluteus maximus and mid-thigh muscle was significant after BMI adjustment between the muscle area comparison (*P*_Gmax-men_ = 0.0453; P_midthigh-women_ = 0.0407). The specific adjustment results are shown in **Table 3**.

**Table 2:** Correlation of variables with BMI and age.

	BeiJing Men	KunMing Men	BeiJing Women	KunMing Women
	Age BMI	Age BMI	Age BMI	Age BMI
**G.MaxM density (HU)**	-0.12 (0.22); -0.35 (**)	-0.32 (*); -0.238 (*)	-0.28 (**); -0.27 (**)	-0.28 (*); -0.29 (**)
**G.MaxM area (cm^2^)**	-0.21 (*); 0.39 (**)	-0.20 (0.07);-0.10 (0.36)	-0.15 (0.04); 0.51 (**)	-0.08 (0.38); 0.51 (**)
**Midthigh muscle density (HU)**	-0.30 (*); -0.13 (0.17)	-0.22 (*); -0.02 (0.86)	-0.36 (**); -0.10 (0.18)	-0.25 (*); -0.16 (0.06)
**Midthigh muscle area (cm^2^)**	-0.31 (*); 0.44 (**)	-0.21 (0.06)**;** 0.006 (0.96)	-0.34 (**); 0.55 (**)	-0.23 (*); 0.45 (**)
**G.Med/MinM density (HU)**	-0.34 (**); -0.13 (0.17)	-0.31 (*); -0.17 (0.12)	-0.36 (**);-0.08 (0.26)	-0.48 (**); -0.12 (0.16)
**L2 trunk muscle density (HU)**	-0.32 (**); -0.17 (0.09)	-0.34 (*); -0.17 (0.13)	-0.3 (**); -0.23 (*)	-0.34 (**); -0.38 (**)
**L2 trunk muscle area (cm^2^)**	-0.28 (*); 0.57 (**)	-0.31 (*); 0.008 (0.94)	-0.2 (*); 0.54 (**)	-0.18 (*); 0.42 (**)

Data presented as correlation coefficient R and (P value)*P <.05 **P <.001. The values of P < 0.05 were marked in bold.

**Table 3:** Difference of variables between Beijing and Kunming after adjusted factors.

	G.MaxM density (HU)	G.MaxM area(cm^2^)	Midthigh density (HU)	Midthigh area(cm^2^)	G.Med/MinM density (HU)	L2 trunk muscle density (HU)	L2 trunk muscle area(cm^2^)
**Adjusted age factor**							
**BJM-KMM**	**P<0.0001**	P=0.3431	P=1	P=0.1108	P=1	**P<0.0001**	P=0.5117
**BJW-KMW**	**P=0.0006**	P=0.4202	P=1	P=0.7357	P=0.0769	**P<0.0001**	P=0.1526
**Adjusted BMI factor**							
**BJM-KMM**	**P=0.0001**	**P=0.0453**	P=1	P=0.5913	P=1	**P<0.0001**	P=0.4415
**BJW-KMW**	**P=0.0134**	P=0.1722	P=1	**P=0.0407**	P=0.1613	**P<0.0001**	P=0.5956
**Adjusted age and BMI factors**							
**BJM-KMM**	**P=0.0009**	P=0.1239	P=1	P=0.3019	P=1	**P<0.0001**	P=0.8350
**BJW-KMW**	**P=0.0375**	P=0.2590	P=1	P=0.1020	P=**0.0116**	**P<0.0001**	P=0.8197

Data presented as (mean ± SD) BJM, Beijing Men; BJW, Beijing Women; KMM, Kunming Men; KMW, Kunming Women. The values of P < 0.05 were marked in bold.

### Age Stratified and Influence Factors Adjusted Results

To further explore age-related muscle degeneration, we stratified the analyses by age with a cut point of 70 years (**Table 4**). In the younger group, there was no difference in age between the Beijing and Kunming groups (*P*>0.05), but the BMI difference was statistically significant (*P*_men_=0.0148, *P*_women_ **<**0.0001). After age and BMI adjustment, the L2-trunk muscle density of the male and female Kunming groups was significantly higher than those of the Beijing population both in the younger and older groups (*P*_adjusted_< 0.0001). However, after adjustment for age and BMI the density difference in the gluteus maximus muscle disappeared in the younger women as well as the older men (younger women group: *P*_adjusted_ = 0.0689; older men group: *P*_adjusted_ =0.0667).

**Table 4:** Difference of variables between Beijing and Kunming after age-stratified and variables adjusted.

Younger group[60~70]	Beijing men	Kunming men	P/P_a_ value	Beijing Women	Kunming Women	P/P_a_ value
	N=73	N=58		N=142	N=103	
Age (years)	65.67 ± 3.01	64.84 ± 3.17	**P=0.12**	64.77 ± 3.01	64.27 ± 3.18	P=0.20
BMI (kg/m^2^)	25.40 ± 2.65	24.03 ± 3.27	**P=0.0148**	25.53 ± 3.20	23.08 ± 3.68	**P<0.0001**
G.MaxM density (HU)	35.52 ± 6.71	40.17 ± 4.37	**P< 0.0001** **P_a_ = 0.0005**	32.52 ± 6.16	35.00 ± 5.44	**P=0.0043** P_a_ = 0.0689
G.MaxM area (cm^2^)	44.29 ± 8.01	45.87 ± 7.17	P=0.3638 P_a_ =0.1068	37.89 ± 6.05	37.17 ± 6.29	P=0.3502 P_a_ =0.2115
G.Med/MinM density (HU)	43.46 ± 4.06	44.21 ± 3.53	P=0.2686 P_a_ = 0.7337	41.99 ± 4.04	41.22 ± 3.92	P=0.246 **P_a_ =0.0141**
Midthigh muscle density (HU)	46.67 ± 3.48	47.05 ± 2.07	P=0.7384 P_a_ = 0.8827	44.17 ± 3.76	44.55 ± 2.96	P=0.7011 P_a_ = 0.8330
Midthigh muscle area (cm^2^)	127.37 ± 22.93	122.76 ± 18.44	P=0.073 P_a_ =0.4905	95.96 ± 14.50	94.94 ± 12.82	P=0.5687 P_a_ 0.0900
L2 trunk muscle density (HU)	30.90 ± 4.09	38.13 ± 4.08	**P< 0.0001** **P_a_=<0.0001**	28.13 ± 3.55	32.37 ± 4.58	**P< 0.0001** **P_a_ <0.0001**
L2 trunk muscle area (cm^2^)	129.43 ± 17.25	129.60 ± 20.98	P =0.8784 P_a_ =0.4249	92.61 ± 13.41	89.47 ± 11.98	P =0.0921 P_a_ =0.9195
**The older group(>70years)**	**N=34**	**N=25**		**N=51**	**N=32**	
Age (years)	78.0 ± 3.84	74.96 ± 3.85	**P=0.0028**	75.80 ± 3.21	75.34 ± 3.87	P=0.33
BMI (kg/m^2^)	24.37 ± 2.44	23.74 ± 2.72	P=0.39	24.69 ± 2.64	24.55 ± 3.95	P=0.97
G.MaxM density (HU)	34.24 ± 6.18	37.48 ± 3.88	**P=0.0272** P_a_=0.0667	28.55 ± 5.64	31.622 ± 6.52	**P=0.0432** **P_a_ = 0.0297**
G.MaxM area (cm^2^)	40.57 ± 7.11	41.88 ± 7.32	P=0.3989 P_a_ =0.8109	35.53 ± 6.65	35.79 ± 5.64	P=0.6233 P_a_ =0.8999
G.Med/MinM density (HU)	41.15 ± 3.42	41.80 ± 4.08	P=0.4075 P_a_ =0.7323	38.69 ± 4.21	37.14 ± 4.81	P=0.2066 P_a_ = 0.0898
Midthigh muscle density (HU)	44.68 ± 3.65	45.46 ± 3.27	P=0.2025 P_a_ =0.5807	41.59 ± 3.45	42.62 ± 3.42	P=0.2693 P_a_ =0.2378
Midthigh muscle area (cm^2^)	115.34 ± 18.43	113.62 ± 18.29	P=0.7706 P_a_ =0.4735	85.57 ± 11.58	87.62 ± 13.54	P=0.6601 P_a_ =0.4871
L2 trunk muscle density (HU)	28.03 ± 3.68	35.53 ± 4.13	**P<0.0001** **P_a_ <0.0001**	25.27 ± 3.54	28.72 ± 5.79	**P=0.0032** **P_a_= 0.0006**
L2 trunk muscle area (cm^2^)	118.32 ± 17.50	116.32 ± 14.58	P=0.83 P_a_ =0.2749	84.08 ± 11.36	86.10 ± 11.46	P=0.1633 P_a_ =0.4279

Data presented as (mean ± SD) P-value: unadjusted P-value; P_a_ value: age and BMI adjusted P-value. The values of P < 0.05 were marked in bold.

## Discussion

To our knowledge, this is the first study to compare the muscle characteristics (density and area) between people living in high- and low-altitude areas using quantitative CT scans. After adjustment for age and BMI, the density of L2-trunk muscle and gluteus maximus muscle in people living at high altitude (Kunming) were significantly higher. We also found that BMI decreased with the increased altitude. All the results above indicate that people living with higher attitude are slimmer and have better muscle quality. This critical finding may be valuable for the update of the international consensus statements of sarcopenia such as those from the Asian Working Group for Sarcopenia (AWGS) in 2019 and from the European Working Group on Sarcopenia in Older People (EWGSOP) in 2018 (16, 17). The role of CT or MRI to measure muscle size as a diagnostic criterion of sarcopenia has not been well specified. The associations of muscle mass and size with muscle function are weak. In this study we found the altitude affected the muscle density (muscle quality) but not the size, which high value the use of muscle quality by CT or MRI better characterizes muscle function and may assign a more domain role to CT and MR in the diagnosis of sarcopenia in treatment planning and monitoring response to treatment. The findings in our study provide evidence that muscle density assessed by CT imaging may be a sensitive screening tool for sarcopenia at different altitudes.

Recent data indicated that the incidence of hip fractures is associated with increased altitude (1). Muscle function is important in preventing falls and related osteoporotic fractures (10). To date, the relationship between muscle density or muscle size with attitude is still unclear. In our study, both for men and women, the muscle density of L2 trunk muscle and gluteus maximus muscle in the Kunming population was higher than that in Beijing, and those results were independent of BMI and age. However, no significant difference was observed in the muscle area. This density difference is believed to be associated with fatty infiltration of skeletal muscle., Muscle density in this study was measured by CT threshold segmentation. After removing the influence of fat infiltration in muscle space, factors such as fat infiltration in muscle cells and myoglobin concentration may be the main influencing factors affecting muscle density. A study by Chia et al. found that total body fat mass measured by DXA was significantly decreased and lean mass increased in ten young male swimmers after 3-weeks of training at an altitude of 2,300 m, while there was no change in body composition in eight male control subjects who resided at sea level for the same period (13). In addition, the total hemoglobin was simultaneously increased significantly in the skeletal muscle. Similar results were obtained in previous animal experiments (18). Based on the findings of previous studies, the decrease of fat content under hypoxia may be related to the following factors: 1) sympathetic power is significantly elevated under hypoxia, which might influence the regulation of body composition by altering blood distribution among adipose and muscle tissues. This change is followed by energy fuel redistribution and increased insulin delivery toward skeletal muscle (19); 2) hypoxic regulation of human skeletal muscle mitochondria. A study reported that mitochondrial volume density increased after twenty-eight days of acclimatization at 3,454 m (20). However, the current interpretation of the effect of hypoxia on skeletal muscle mitochondria is inconsistent or even contradictory and needs to be further clarified; 3) Effect of endocrine metabolism, particularly glucose homeostasis and lipid metabolism. Interestingly, studies found that individuals living at higher altitudes have lower fasting glycemia and better glucose tolerance compared with those who live near sea level (21). In short, contrary to our initial hypothesis, muscle density increases with altitude. Meanwhile, with an increase in altitude, bone mass decreases and fragility increases (1). The high incidence of hip fracture in the plateau area might be the consequence of muscle and bone interaction, but the specific regulatory mechanism is not clear. We hypothesize that the difference between the muscle measurements in Kunming and Beijing might be a self-protection compensation mechanism for the body to resist bone loss under the hypoxic environment. Further researches, however, are needed. Trunk muscle density was the most sensitive variable in our research. Compared to gluteus and mid-thigh muscles, the density of trunk muscle was a more sensitive parameter indicating that people living with higher attitude have better muscle quality, independent of BMI and age. This also indicated that the trunk muscle may be somewhat different from the other muscles in its response to hypoxia or underlying obesity, but the mechanism is unclear. A previous study showed similar results that hepatic steatosis predicted psoas muscle fat content independent of BMI (22). In addition, low trunk muscle density has proved to be associated with poor balance, lower and faster declines in functional capacity in older adults (23, 24). These results suggest that trunk muscle density may have potential value in future fat deposition assessment and sarcopenia diagnosis for the plateau area residents.

In this study, we observed the interesting finding of decreased BMI but no changes in muscle size with increased attitude, which indicated that people living in Beijing at low attitude have more fat depots in the body, and the male Beijing group showed a larger waist circumference as expected. De Carvalho et al. found that abdominal obesity was associated with accelerated muscle strength decline in men (25). His study may provide a strong reference for the interpretation of our findings. Tissue-specific lipid partitioning changes could lead to altering the distribution of fat in the body (26). The location of triglyceride (TG) storage has important metabolic consequences. Visceral fat was also found to be strongly associated with elevated triglycerides levels and fatty infiltration of muscle tissue (27, 28). Our findings show that high attitude impacts fat deposition, namely by decreasing the fat in the abdomen and intramyocellular lipids.

Bodyweight reduction is an inevitable consequence of chronic hypoxic exposure (5). In our study, people living at higher altitude were found to have lower BMI, consistent with the study of Ye et al. (8). Previous studies on the relationship between muscle and altitude mainly focused on the mass assessed by DXA or BIA but the results are inconsistent. Some reports showed that skeletal muscle mass decreases with increased altitude, while in others it was unchanged or increased (8, 13, 20). The variations of BMI may be the main reason for this discrepancy. The decrease of muscle mass with increased altitude in some studies may be caused the decrease in BMI. Meanwhile, due to the obvious influence of BMI, the muscle mass of different studies may not be directly compared. Furthermore, body mass by DXA or BIA may not fully reflect the underlying pathophysiology of muscle strength and related functional outcomes. These findings suggest that muscle mass may not be a appropriate index to evaluate the effect of hypoxia on muscles. Muscle strength and physical performance have come to be recognized as deserving more attention in the musculoskeletal field study (17, 29). Muscle density may be a better quantitative index in this case (12). Nevertheless, there remains a lack of research on muscle density and the corresponding reference density value in the hypoxia environment. This study was undertaken to provide a reference for further clinical research and mechanism exploration in this field.

This study has several limitations. A major limitation is a cross-sectional design and a limited sample size. Another limitation is the lack of evaluation of physical activity (PA) and local eating habits for both cohorts. However, the respective dietary habit surveys from Beijing and Kunming suggested the diet type and energy intake in the old population were not considered to be significant (30, 31). Moreover, previous studies showed that the PA differences were mainly concentrated between urban and rural areas and all subjects included in this study were elder urban residents (32, 33), so the differences in PA between the two groups of our study were hypothesized to be relatively small. Further, a large epidemiologic study in China found that the total average physical activity level was obviously lower for the 60 to79 years old population compared to young age groups (33), and the main types of PA were occupational PA (62%), followed by domestic PA (26%) and leisure-time PA (4%). The subjects in this study were over 60 years old and most of them were retired, the domestic and leisure time PA becoming the main part. A study showed 85.4% of the elderly (over 60 years) in China did not engage in leisure-time exercise (34). These results indicate that the PA difference between the two groups in this study might be small and may have little influence on interpreting the results in this study. What’s more, physical activity or exercise increases muscle size (35) which did not differ between Beijing and Kunming subjects. Thus, in our cohort, there was probably no significant difference in PA behaviors and eating habits between the two groups.

## Conclusion

In conclusion, people living in the Yunnan plateau region have a higher density of L2-trunk muscle and gluteus maximus muscle compared with those living in a low altitude area such as Beijing. In addition, our study provides reference data for muscle density of the plateau population for the first time.

## Data Availability Statement

The original contributions presented in the study are included in the article/supplementary material. Further inquiries can be directed to the corresponding authors.

## Ethics Statement

The studies involving human participants were reviewed and approved by Beijing Jishuitan Hospital (No:201512-02) and The first people’s hospital of Yunnan province (No.KHLL2021-KY056). The patients/participants provided their written informed consent to participate in this study. Written informed consent was obtained from the individual(s) for the publication of any potentially identifiable images or data included in this article.

## Author Contributions

LL and XC designed the study, developed the theoretical framework, and supervised the project. XL, LW, MG, and GW analysed the results and drafted the manuscript. KT, JinY, WS, and JingsongY completed the data collection, such as questionnaire information collection, scanning and data input. All authors contributed to the article and approved the submitted version.

## Funding

This work was supported by the National Natural Science Foundation of China [grant number: 81901718, 81771831], The Beijing Hospitals Authority Clinical Medicine Development of Special Funding Support [grant number: ZYLX202107] and Yunnan “Ten thousand people plan”-famous doctor special project [grant number: YNWR-MY-2019-011]. The funders had no role in study design, data collection, and analysis, or preparation of the manuscript.

## Conflict of Interest

The authors declare that the research was conducted in the absence of any commercial or financial relationships that could be construed as a potential conflict of interest.

## Publisher’s Note

All claims expressed in this article are solely those of the authors and do not necessarily represent those of their affiliated organizations, or those of the publisher, the editors and the reviewers. Any product that may be evaluated in this article, or claim that may be made by its manufacturer, is not guaranteed or endorsed by the publisher.
